# TV medical dramas: assessing the portrayal of public health in primetime

**DOI:** 10.3389/fpubh.2024.1432528

**Published:** 2024-07-24

**Authors:** Daniela Zago, Peter Cautero, Enrico Scarpis, Erika Pompili, Gianluca Voglino, Roberta Siliquini, Silvio Brusaferro, Laura Brunelli

**Affiliations:** ^1^Dipartimento di Medicina, Università di Udine, Udine, Italy; ^2^Dipartimento di Scienze della Sanità Pubblica e Pediatriche, Università di Torino, Torino, Italy; ^3^AOU Città della Salute e della Scienza di Torino, Torino, Italy; ^4^SOC Accreditamento, Qualità e Rischio Clinico, Azienda Sanitaria Universitaria Friuli Centrale, Udine, Italy

**Keywords:** medical drama, public health, health promotion, infectious risk management, health communication

## Abstract

**Introduction:**

The influence of medical dramas could extend beyond the realm of entertainment and potentially strengthen/orient the knowledge, attitudes and hopefully practice of health professionals and the public, despite often depicting unrealistic medical outcomes and scenarios.

**Methods:**

This study examined the portrayal of public health issues in two popular international medical series, “Grey’s Anatomy” and “House, MD,” selected for their awards and viewership ratings. Individual episodes were double-rated by clinicians for patient characteristics, public health issues, and infection risk management.

**Results:**

94 episodes with 286 clinical cases were analyzed. A wide range of conditions and pathologies were depicted, with a focus on acute clinical events, trauma and mental disorders, which contrasts with real-life causes of hospitalization and highlight the dramatization in these series. Public health issues such as organ donation and substance abuse were frequently addressed, but prevention and health promotion received little attention. Proper use of personal protective equipment was mostly observed, although hand hygiene was underrepresented.

**Conclusion:**

The study highlights the dual role of medical dramas as an entertainment medium and a potential educational tool. While they can raise awareness and encourage healthier behavior, their portrayal of medical practices and patient care often deviates from reality and can create unrealistic expectations. The influence of these dramas also extends to viewers’ perceptions of healthcare and medical professionals, underscoring the need for accurate and responsible portrayal of health issues in the media.

## Introduction

The need for health information and literacy is constantly increasing (1), and in recent years the tools used by the population have changed and evolved rapidly, mainly thanks to the introduction of the Internet (2). Despite the proliferation of web tools, traditional media such as television continues to be used by a large proportion of the population (3), although this distinction is becoming increasingly blurred with the popularity of on-demand streaming services. Medical dramas are one component of this media. This term refers to a subgenre of dramas—fictional serial products—that are characterized by a medical setting. This type of product has evolved over the years from a disinterested portrayal of the doctor to one that focuses on the private lives of the professionals portrayed (4). At the same time, thanks to their ever-increasing success, these programs have sought to more accurately reflect the realities portrayed, but without sacrificing entertainment (5).

Several studies have analyzed the impact of this type of entertainment and its various developments. On the one hand, some studies have observed how medical dramas can be used as a means of training healthcare professionals, in particular to stimulate activities in the classroom (6), also thanks to the representation of hidden curricula (7). On the other hand, another part of the literature has focused on the quality of the representation of the different medical actions. These studies have focused on a wide range of areas, such as surgery (8), radiology (9) and emergency medicine, especially cardiopulmonary resuscitation (10, 11). These studies have shown that the representations are often unrealistic and show results that do not correspond to reality, potentially altering patient expectations. In addition to these critical aspects, there is a sometimes-unflattering portrayal of healthcare professionals that is characterized by racism, moral corruption and interpersonal conflicts (12), which threatens to tarnish the image of the profession. It should also be borne in mind that a significant proportion of the population still has a limited level of health literacy and therefore may not be able to judge the quality of representation (13). Nonetheless, it has been observed how medical dramas can influence both healthcare professionals and aspiring healthcare professionals (14–16).

In recent years, it has become increasingly clear how important it is to address the current public health challenges of our time—from antibiotic resistance (17) to the prevalence of cardiovascular risk factors (18), from environmental pollution (19) to epidemic preparedness (20) and prevention of healthcare-associated infections (21). To address these issues, but also to promote population health and prevent both communicable and non-communicable diseases, special attention must be paid to education as a public health objective, and tools such as medical dramas can be useful in this regard. To our knowledge, no study has examined the quality of public health portrayal in the media. The aim of this study was therefore to assess the nature, frequency and accuracy of the portrayal of public health issues in several well-known medical dramas.

## Materials and methods

### Selection of TV series

TV series were identified that fall into the “medical drama” category. To identify internationally broadcast TV series, American or Canadian TV series or international productions that included one of these countries were selected. Only English-language TV series that aired after 1990 were selected. To assess the influence of these TV series, the number of awards that each series (or individual professionals for their work on that series) had won was taken into account. The awards won by the series at 15 different award ceremonies, chosen for their notoriety and representativeness of the various actors in the world of television. In addition, the nominations of each series (or individual professionals for their work on that series) were taken into account. The awards considered are:

- Emmy Awards: presented since 1949 by the “Academy of Television Arts & Sciences” to recognize outstanding achievements in the world of television. It is considered one of the most prestigious international awards, on a par with the Oscar (film), the Grammy (music) and the Toni Award (theater). Both awards, which are presented as the “Primetime Emmy Awards,” “Daytime Emmy Awards” and “Creative Arts Emmy Awards,” were considered for this study.
- Golden Globe Awards: presented annually by the “Hollywood Foreign Press Association” to recognize the best achievements in the world of film and television.
- Young Artist Awards: presented annually by the “Young Artist Foundation” to honor the best young artists.
- People Choice Awards: voting is done online by fans and aims to recognize the promotion of pop culture.
- Satellite Awards: presented annually by the “International Press Academy” to honor the best in the entertainment industry.
- Peabody: presented by the College of Georgia to individuals who have made outstanding contributions to public service in the areas of radio and television news, film and television documentary, youth education programs and entertainment.
- Teen Choice Awards: online voting by teens for popular music, movies, and television.
- TCA Awards: presented by the Television Critics Association, the awards recognize excellence in television each summer in 11 categories.
- Writers Guild of America Awards: since 1949, the two American screenwriters’ unions have organized an awards ceremony, the Writers Guild of America Awards, in three categories.
- Directors Guild of America Awards: this award is presented annually by the Directors Guild of America, the union representing American directors. It is not only one of the most prestigious awards for directors but is also considered the most reliable indicator of an Oscar win.
- Producers Guild of America Awards: a festive event to honor the best employees of film and television producers. The event was established in 1990 by the Producers Guild of America under the direction and support of President Leonard B. Stern.
- Screen Actor Guild Awards: an award presented annually by the Screen Actors Guild for the best performances of its members.
- NAACP Awards: an award presented annually by the National Association for the Advancement of Colored People (NAACP) in recognition of the work of Blacks in the arts.
- GLAAD Media Awards: an annual award established in 1990 by the Gay & Lesbian Alliance Against Defamation to recognize individuals and productions in the entertainment industry that help present a truer and more accurate picture of the LGBT community and the issues that affect their lives.
- ALMA Awards: honors Latino performers (actors, musicians, singers, and fashion designers) who promote positive representation of Latinos on television and in entertainment. In North America, the ALMA Award is often referred to as the “Academy Awards of Latin America.”

The television series were then ranked according to the number of awards won. In the event of a tie, according to the number of nominations received but not won. If there was a tie based on the number of awards won and the number of nominations received, they were ranked according to the most recent series. Secondly, TV series that are broadcast daily or not weekly were excluded. Based on these criteria, two medical dramas were selected for the study, *Grey’s Anatomy* (GA) and *House, M.D.* (DH), as they aired primetime in the U.S. between 2010 and 2019 and in Italy between 2011 and 2019 and are still available on DVD or paid platforms. Also due to time constraints, we have limited our analysis to just two seasons.

### Evaluation of TV series

The research group developed an *ad hoc* checklist to evaluate each episode of the selected TV series. The unit of analysis was set at the individual episode level, with every patient for whom an actor was selected or for whom the characters work (even if they are never seen), every public health issue the characters talk about or deal with, and every procedure requiring patient contact as elements to be evaluated. If more than one patient was depicted in an episode, all patients were included in our dataset. The variables collected were divided into three main categories: (1) characteristics of the patient and their care during the episode, (2) public health issues, which are considered conditions that negatively impact the health of a population and can be prevented or mitigated by public health interventions (22), 2.1 health prevention and promotion, 2.2 patient safety and infection risks. The full list of variables includes:

1. Patient age (stated or estimated), sex and ethnicity. The research group also evaluated other variables that may have influenced patient care, such as sexual orientation, gender identity, socioeconomic status, religious orientation, health insurance, waiting time for care (estimated elapsed time to visit/acceptance expressed as order of magnitude -minutes, hours, days, months). We also assessed the reason for seeking health care, type of service utilized, initial and final diagnosis during the episode, tests performed, medications administered, outcome, and need for recovery.
2. Public health issues
  - 2.1 Health prevention and promotion: immunizations, screening programs, smoking and alcohol habits/abuse, healthy diet, physical activity, sleep hygiene, sexually transmitted diseases, substance abuse; blood/organ donation, vanguard of care, adherence to work schedules, violence against health professionals in the workplace, patient safety (correct patient identification, effective communication, surgical patient safety) (22, 23);
  - 2.2 Patient safety and infection risks: for each procedure performed, data were collected on the type of procedure, use of gloves, disposable gowns and surgical masks, and hand hygiene performed (24). The original checklist was tested during the pilot phase for some episodes, and changes were made to the checklist or information was added in order to compile it correctly. The final version of the checklist is attached as a Supplementary material to this manuscript.

The evaluation of the TV series episodes was conducted in 2019 between March and August. Ninety-four episodes were evaluated: 49 (52.1%) from GA and 45 (47.9%) from DH. Specifically, season 14 (24 episodes) and season 15 (25 episodes) of GA and season 7 (23 episodes) and season 8 (22 episodes) of MD were analyzed; a total of 70.5 h of video were viewed. To minimize bias and increase the reliability and completeness of data collection, each episode was analyzed by two clinicians in blind using the above checklist; each clinician viewed the episode and recorded the data independently without knowing the colleague’s interpretation; the data were then compared and any conflicting information recorded by the researchers was discussed until consensus was reached. Patients or the public were not involved in the design, conduct, reporting dissemination plans of our research.

### Data analysis

Diseases identified as initial and final diagnoses were classified according to the ICD-9-CM manual. Procedures were categorized into 12 groups (medications; surgical procedures; urinary catheterization; venous and arterial, peripheral and central catheters; assisted ventilation; hemodialysis; prosthetic heart valve; pacemakers; vascular prostheses; ventricular shunts; biopsy; medical examination; other) and surgical procedures were then further grouped into clean, clean-contaminated, contaminated, dirty/infected. For the analysis of the adequacy of personal protective equipment, procedures were grouped in three main categories: clean, intermediate, and surgical. Hand hygiene was considered necessary for all procedures; the use of gloves was considered necessary for intermediate procedures; the use of gloves, disposable gown and surgical mask was considered necessary for surgical procedures. Descriptive analyses were performed to characterize the clinical cases analyzed. The continuous variables were expressed as median value and interquartile range, the qualitative variables as absolute value and percentage. All statistical analyses were performed using STATA IC14 software.

## Results

### General characteristics

A total of 286 clinical cases were identified and subsequently analyzed: 216 (76%) from the GA series and 70 (24%) from the DH series. For all episodes, all represented clinical cases were reported and analyzed: on average, there were 3 clinical cases per episode (minimum 1; maximum 13). Most patients were middle-aged Caucasian (71.0%) and slightly more often male (51.7%). In most cases, sexual orientation, socioeconomic status, religious orientation, availability of health insurance, and waiting time for treatment had no effect on patient treatment. The general characteristics of the patient population are described in Table 1.

**Table 1:** Distribution of the general characteristics of the shown clinical cases.

Patient characteristic	Total	%
Age group neonatal (0–1 y) pediatric (1–16 y) youth (17–30 y) middle-aged (31–65 y) older adult (>65 y) missing	8 46 54 148 28 2	2.8 16.1 18.9 51.7 9.8 0.7
Sex male female missing	148 136 2	51.7 47.6 0.7
Ethnicity Caucasian African-American Asian Middle Eastern Hispanic missing	203 49 20 3 5 6	71.0 17.1 7.0 1.1 1.7 2.1
Effect on care due to sexual orientation yes no missing	1 283 2	0.3 99.0 0.7
Effect on care due to gender identity yes no missing	1 283 2	0.3 99.0 0.7
Effect on care due to socioeconomic status yes no missing	5 279 2	1.7 97.6 0.7
Effect on care due to religious orientation yes no missing	6 278 2	2.1 97.2 0.7
Effect on care due to health insurance yes no missing	4 280 2	1.5 97.8 0.7
Effect on care due to waiting time for care yes no missing	3 280 3	1.1 97.8 1.1

In most cases (*n* = 210, 73.4%), the clinical event occurred acutely, whereas the chronic or exacerbated form of clinical onset was less represented (*n* = 38, 13.3% and *n* = 20, 7.0%, respectively); in 18 cases, information on the onset of the clinical condition was missing (6.7%). Trauma and poisoning were the most common initial diagnoses with 93 cases (31.5%), followed by unclear signs and symptoms (*n* = 80, 27.1%), mental disorders (*n* = 23, 7.8%) and neoplasms (*n* = 20, 6.8%). Apart from cancer and congenital malformations, the original diagnoses were confirmed during the episode in less than 50% of cases. Infectious diseases were the first diagnosis in only two cases, which later turned out to be wrong, while they were the final diagnosis in 24 cases, although they had not been recognized at the beginning of the episode. Further details of the clinical conditions at initial and final diagnosis in the TV medical dramas studied can be found in Table 2.

**Table 2:** Distribution of initial and final diagnoses of the proposed clinical cases, classified by the ICD-9-CM and their concordance in the single episode.

Clinical situation (ICD-9-CM)	Initial diagnosis*	Final diagnosis	Initial-final diagnoses concordance^§^
	n (%)	n (%)	n (%)
Injury and poisoning	93 (31.5)	28 (11.3)	18 (19.4)
Symptoms, signs, and ill-defined conditions	80 (27.1)	8 (3.2)	4 (5.0)
Mental disorders	23 (7.8)	13 (5.2)	2 (8.7)
Cancer	20 (6.8)	36 (14.5)	12 (60.0)
Diseases of the digestive system	12 (4.1)	17 (6.9)	1 (8.3)
Diseases of the circulatory system	11 (3.7)	28 (11.3)	3 (27.3)
Complications of pregnancy, childbirth, and the puerperium	11 (3.7)	7 (2.8)	3 (27.3)
Diseases of the nervous system and sense organs	10 (3.4)	24 (9.7)	3 (30.0)
Diseases of the respiratory system	9 (3.1)	20 (8.1)	2 (22.2)
Diseases of the blood and blood-forming organs	6 (2)	12 (4.8)	0 (0.0)
Diseases of the musculoskeletal system and connective tissue	6 (2)	9 (3.6)	0 (0.0)
Diseases of the genitourinary system	5 (1.7)	5 (2)	1 (20.0)
Endocrine, nutritional and metabolic diseases, and immunity disorders	3 (1)	8 (3.2)	0 (0.0)
Infectious and parasitic diseases	2 (0.7)	24 (9.7)	0 (0.0)
Diseases of the skin and subcutaneous tissue	2 (0.7)	3 (1.2)	0 (0.0)
Congenital anomalies	2 (0.7)	6 (2.4)	1 (50.0)
Total	295	248	50

*more than one diagnosis was possible for the same patient; ^§^the percentage was calculated based on the number of initial diagnoses.

### Public health issues

At least one public health issue was addressed in 57 (61%) episodes (86 cases in total): 60 (70%) in GA and 26 (30%) in DH. Compared to the number of clinical cases treated, public health is less represented in GA than in DH (27.7% vs. 37.1%). The most frequently addressed public health issues in both series were blood and organ donation and drug and alcohol abuse, while disease prevention (i.e., immunization, screenings) was scarce, and sleep hygiene was not mentioned. Public health promotion issues were covered more frequently in the GA seasons examined (37/49 episodes) than in the DH seasons (20/45 episodes). The issues covered are listed in Table 3.

**Table 3:** Distribution of the public health issues covered in the proposed clinical cases.

Public health issue*	Total (*N* = 86)	GA (*N* = 60)	DH (*N* = 26)	Form of representation of each public health issue (n; %)
	n (%)	n (%)	n (%)	
Blood and Organ donation	28 (33)	18 (64)	10 (36)	Bone marrow or solid organ transplantation and possible complications (13; 46%) Requirements for inclusion/exclusion on transplant waiting lists (6; 21%) blood donation awareness (4; 14%) cutting-edge care (3; 11%) blood donation awareness (2; 7%)
Drug abuse	23 (27)	17 (74)	6 (26)	abuse by minors (7; 30%) drug abuse and withdrawal syndrome (6; 26%) abuse by a health care professional (5; 22%) abuse for pain management (3; 13%) support groups (2; 9%)
Alcohol abuse	15 (17)	12 (80)	3 (20)	alcohol-related pathologies (6; 40%) abuse by a health care professional (5; 33%) support groups (3;20%) abuse by minors (1; 7%)
Sexually transmitted diseases	5 (6)	1 (20)	4 (80)	syphilis (4; 80%) gonorrhea (1; 20%)
Healthy eating	4 (5)	3 (75)	1 (25)	use of healthy food (3; 75%) discouraged red meat use (1; 25%)
Physical activity	4 (5)	4 (100)	0 (0)	cardiovascular prevention (4; 100%)
Immunizations	3 (3)	1 (33)	2 (66)	neonatal vaccinations (1; 33%) vaccinations in immunocompromised (1; 33%) chickenpox in an unvaccinated household contact (1; 33%)
Screening programs	3 (3)	3 (100)	0 (0)	prenatal screening test (2; 67%) pre-therapy screening (1; 33%)
Smoking	1 (1)	1 (100)	0 (0)	Smoking abstinence requirement for organ transplantation (1; 100%)
Patient safety	92 (32)	74 (80)	18 (20)	cutting-edge medical care (37; 40%) surgical patient safety (20; 22%) working hours (17; 19%) effective communication (9; 10%) workplace violence (7; 8%) patient identification (2; 2%)

*more than one issue was possible for the same case.

In the episodes analyzed, a total of 239 procedures were performed, 160 (66.9%) in the GA and 79 (33.1%) in the DH. Measured by the number of clinical cases treated, the procedures are more represented in the DH than in the GA (79/70 vs. 160/216). According to the main categories identified to assess the appropriateness of personal protective equipment, 64.0% of procedures were classified as surgical (*n* = 153), 18.4% as intermediate (*n* = 44), and 17.6% as clean (*n* = 42). Regardless of the type of procedure performed, hand hygiene was shown on the screen 25 times (in 10.5% of cases). The use of gloves was found to be correct in 182 (92.4%) of the 197 cases where this was required, while the disposable gown was used in 134 (87.6%) of the 153 cases. The surgical mask was used correctly in 124 (81%) of the 153 procedures.

## Discussion

This study examined the types of clinical cases depicted in some episodes of two medical dramas, which are among the most popular programs, with particular attention to issues of prevention and health promotion. In particular, 94 episodes were observed and for each clinical case, the general characteristics, public health portrayal and use of infectious disease prevention practices were assessed. Our analysis revealed that a wide variety of clinical cases covering all categories of the ICD-9 classification were addressed in the different episodes, while public health topics were limitedly represented. In fact, health promotion topics were inadequately addressed (in just over half of the episodes), with important public health issues such as immunization, screening programs and smoking control covered in less than 5 % of the cases. Infection risk prevention measures were also inadequately addressed, with hand hygiene practiced in only 10 % of the required cases, despite its critical importance. Medical dramas have been a staple of television for many years, captivating viewers with their gripping stories and dramatic medical emergencies. However, it should not be forgotten that these shows do far more than entertain, they can also convey important health messages to the public.

### General characteristics

One of the most important ways in which medical dramas can convey health messages is through information, education, and awareness. These shows often explore a wide range of diseases and treatments to give viewers a basic understanding of various health issues. For example, in the television series Grey’s Anatomy, characters often discuss diseases and procedures, shedding light on complex medical issues that the public might not otherwise be aware of. In addition, these shows not only provide in-depth information about complex medical issues, but also have a significant impact on viewers’ behavior, as a 2001 CDC survey found: more than half of regular viewers of medical television shows not only reported learning something new about a disease or health problem, but 34% of regular viewers took one or more actions after hearing about it, such as talking to a friend about it, taking preventive measures, or getting a medical check-up (5).

Another educational aspect of medical dramas is to stimulate curiosity about topics that are often overlooked by the public. Some work has shown how lesser-known diseases suddenly gained prominence in the mainstream media following statements by well-known personalities, as was the case with Ramsay-Hunt syndrome or pancreatic neuroendocrine tumor (25, 26). A tangible example of this effect was provided by Jarrentrup and colleagues in a seminar based on the nature of DH compared to conventional seminars. The students showed a high motivation to learn about rare diseases and they were positively influenced by TV series such as Dr. House to improve their diagnostic and clinical skills. In fact, they attested to excellent diagnostic and therapeutic skills, and in this growth process, they reported an increased learning effect (69.9%), improved concentration (89.7%), increased motivation to participate (88.7%), and greater enjoyment (86.7%) all with statistical significance (all *p* < 0.001) (27). In addition, each episode aims to solve the clinical case presented: it proceeds logically and analytically, especially in the DH series, analyzing signs and symptoms, hypothesizing, and sometimes making mistakes to show how a correct differential diagnosis should be performed in a clinical process. This is also reflected in the poor agreement between initial and final diagnosis, as our results show: for all episodes considered, with the exception of for cancer and congenital anomalies, a correct diagnosis is made in less than one in three patients. Although the cases shown are generally rare, clinical practice corresponds fairly closely to reality. We found that social variables, such as socioeconomic status, and gender had no impact on the clinical case, although it is well documented in the literature that they play a significant role in perpetuating inequalities in access, treatment, and outcomes (28). On the one hand, this may be an attempt to overcome the causes of discrimination and provide an educational message, but on the other hand, it does not provide space to discuss issues that are still very relevant to global public health today.

However, it should be noted that this positive portrayal can lead to unrealistic expectations among patients, creating a gap between the portrayals in TV dramas and reality. For example, shifting attention to neglected diseases could distract from the more common causes of hospitalization in everyday life. The analysis of Italian hospital discharge data from 2019 (i.e., from before COVID-19) shows that the most common reasons for hospitalization were diseases of the cardiovascular system, diseases and disorders of the musculoskeletal system and connective tissue, and diseases and disorders of the respiratory system (29). This reality differs from the portrayals in the TV medical dramas examined, in which injuries and poisoning as well as mental disorders were the most common reasons for hospitalization. The situation portrayed in TV series also differs from the American reality: according to a 2021 study concerning the primary conditions for which patients are actually hospitalized, sepsis, heart failure, osteoarthritis and pneumonia were the result (30). The portrayal of trauma patients in television dramas also differs from reality, particularly in terms of injury recovery (31). Television often exaggerates the immediacy and clarity of traumatic events, presenting a stylized version that does not necessarily correspond to the chaotic and unpredictable nature of actual trauma situations. From the speed of emergency response to the precision of medical procedures, on-screen dramatization tends to oversimplify and compress timelines for narrative effect. Another example is cardiopulmonary resuscitation (CPR), a crucial life-saving technique that is often portrayed on television as a dramatic endeavor with great success. However, the success rate of CPR is significantly lower than that shown on screen (32). The intricacies of effective CPR, the challenges faced by medical professionals and the emotional toll of resuscitation are often overlooked or simplified for dramaturgical reasons. The influence of television entertainment also extends to viewers’ expectations of the standards that apply to the actions of different professionals. This can affect their decision-making processes and influence their willingness to accept or discuss treatment. In addition, television shapes viewers’ attitudes toward healthcare in general and influences their relationship with healthcare and those responsible for healthcare in society (33). The problem of patients’ unrealistic expectations of science and medicine emerges from the analysis of several questions on potential risk factors for workplace violence conducted by Brunelli et al. in an Italian hospital during the COVID-19 vaccination campaign: almost half of the respondents reported having been the victim of an act of violence during their work shift, in some cases even of a physical nature (34).

### Public health issues—health prevention and promotion

Medical dramas can also promote healthy behaviors and lifestyles (i.e., health promotion). Characters in these shows often deal with the consequences of their actions, such as the negative effects of smoking, excessive alcohol consumption, or unhealthy eating. By highlighting these consequences, medical dramas can encourage viewers to make healthier choices in their own lives, which is described by the concept of entertainment-education (E–E), i.e., the way in which entertainment media can be used for educational purposes (35). For example, E–E experiences have been shown to raise public awareness of issues such as breast cancer (36), HIV transmission (37, 38) and alcohol consumption (39). Another interesting experience is that of Leader et al. who showed that high levels of engagement in promotional campaigns correlate with higher HPV vaccination rates, as well as a greater willingness to engage in dialog with family members and health professionals (40).

On the other hand, we found in our study that little attention was paid to prevention and health promotion issues in the episodes analyzed: a topic in this area was covered in just over half of the episodes analyzed overall and specifically in less than a third of the House, MD series. The most frequently covered topics were organ and tissue donation and alcohol and drug abuse; vaccinations, screening programs and tobacco control, on the other hand, received little attention. Looking at this issue in the light of the six Ps for the future of public health (41), Participation is crucial to counter infodemic and misinformation in healthcare and to promote active public involvement. Positive public health outcomes can only be achieved if citizens are actively involved in decision-making, which is facilitated by scientific communication and behavioral science tools. This empowerment approach is effective when it provides tailored messages for different audiences, considering different levels of health literacy.

### Public health issues—patient safety and infection risks

The use of PPE was correct in almost all procedures presented. The use of gloves, gowns and masks was appropriate in more than 80% of cases, both in the selection of PPE according to the type of procedure presented and in its use. In contrast, the hand hygiene procedure was shown in a total of 10% of cases. There could be several reasons for this. Firstly, medical dramas often focus on the main storyline, which usually involves the main characters’ personal and professional affairs, complex medical cases, and interpersonal dynamics. This can lead to procedures that are considered mundane being simplified or underplayed. Secondly, scriptwriters can assume that the audience understands the importance of these procedures without having to show them in detail. Thirdly, episodes of a television series are of limited length and need to focus on elements that contribute to the main narrative. This can lead to the omission of details that might be considered less relevant. Finally, some medical procedures, even if basic, may not be shown in detail to simplify the narrative and focus the viewer’s attention on the main storyline. Nonetheless, it is worth noting that all the episodes analyzed took place in the pre-pandemic period, when awareness of the risk of contracting communicable diseases was perhaps not as pronounced in developed countries.

TV shows often emphasize emotion rather than health prevention or promotion. The focus is not primarily on medical reality, but sometimes serves as a backdrop for narratives of other kinds, such as romantic or relationship stories. This blurring of the line between reality and fiction in TV shows can lead viewers to perceive unreal situations as true (12). Given the relatively accurate portrayal of reality in these programs, there is an opportunity to use them more effectively as a means of communication. They could convey more accurate messages about medical practices and situations, potentially contributing to the public’s understanding of healthcare. The impact of these broadcast on future healthcare professionals and their professional self-image should also be considered. The way healthcare professionals are portrayed in these programs in terms of their commitment, activities, personal lives and relationships and problems could influence the perceptions and expectations of entry-level healthcare professionals. However, there seems to be a lack of information on how viewers’ behavior or attitudes change before and after watching these series. There is also a lack of accurate information on the socio-demographic characteristics of viewers, which could shed light on the different perspectives and reactions of viewers. In addition to considering the impact of medical drama on public perception and the medical profession, there is an urgent need for collaboration between public health experts and media professionals. The goal of this collaboration would be to create messages that are not only scientifically accurate, but also appealing to the public. By combining the expertise of public health professionals with the storytelling skills of media professionals, it becomes possible to strike a balance between entertainment and education and ensure that health-related content is both engaging and informative.

### Limits

Our study has some limitations. Firstly, TV series produced in the U.S. and Canada were considered, which are suitable for this specific context and audience and represent a different health model to that used in Italy. In addition, for each series two seasons were considered, selected according to the criteria initially established, although we are aware that these are not necessarily representative of the entire series. For this reason, based on the results obtained, we plan to expand the selection of series in the future, both in terms of production countries and time periods, focusing, for example, on post-pandemic productions. Indeed, by analyzing more episodes, changes in the data we collected could be detected. In addition, the study could be expanded and improved by including quantitative data analysis and evaluating data on viewer reactions or changes in knowledge and behavior after exposure to these medical dramas. Surveys or focus groups with regular viewers could provide direct evidence of the educational impact of these programs. Finally, the episodes analyzed were recorded before the COVID-19 pandemic. In the future, the impact of the pandemic on these topics, especially in the area of communicable diseases, needs to be further investigated. However, the method used to analyze the TV series could contribute to a further and more comprehensive analysis of these globally and locally produced series.

## Conclusion

In conclusion, medical dramas have the unique ability to entertain while conveying important health messages to the public. These series serve as a platform for education, health promotion and prevention, building empathy, raising awareness of health issues and promoting critical thinking about healthcare decisions. While they do not always provide a completely accurate picture of the health and medical world, it is important to recognize their value as a tool for disseminating health-related information to a wide audience. Therefore, we advocate for greater awareness of public health issues by producers of medical dramas through the involvement of public health experts as advisors. Future analysis should pay particular attention to how the different representations of different racial and ethnic groups, as well as the healthcare systems in place in different countries, can influence their portrayal in the media and subsequent perception by the public.

## Data availability statement

The raw data supporting the conclusions of this article will be made available by the authors, without undue reservation.

## Author contributions

DZ: Writing – original draft, Methodology, Investigation, Formal analysis, Data curation. PC: Writing – original draft, Methodology, Investigation, Formal analysis, Data curation. ES: Writing – review & editing, Methodology, Investigation, Data curation, Conceptualization. EP: Writing – review & editing, Methodology, Investigation, Data curation. GV: Writing – review & editing, Project administration, Methodology, Investigation, Data curation, Conceptualization. RS: Writing – review & editing, Supervision. SB: Writing – review & editing, Supervision. LB: Writing – review & editing, Project administration, Methodology, Investigation, Conceptualization.
