# Supplementary material for: TV medical dramas: assessing the portrayal of public health in primetime
Source: Front Public Health. 2024 Jul 24;12:1432528. doi: 10.3389/fpubh.2024.1432528 (PMC11303220; doi:10.3389/fpubh.2024.1432528)
Supplement: Supplementary file 1 [file Table_1.DOCX]

| **Public health topic** | |
| --- | --- |
| Blood and Organ donation | - yes - no - missing |
| Drug abuse | - yes - no - missing |
| Alcohol abuse | - yes - no - missing |
| Sexually transmitted diseases | - yes - no - missing |
| Healthy eating | - yes - no - missing |
| Physical activity | - yes - no - missing |
| Immunizations | - yes - no - missing |
| Screening programs | - yes - no - missing |
| Smoking | - yes - no - missing |
| Patient safety | - yes - no - missing |

| **Prevention of Infectious Diseases** | |
| --- | --- |
| Type of procedure | *free field* |
| Gloves | - yes - no - missing |
| Mask | - yes - no - missing |
| Gown | - yes - no - missing |
| Hand hygiene | - yes - no - missing |
